# Optical frequency division

**DOI:** 10.1093/nsr/nwz209

**Published:** 2019-12-19

**Authors:** Yuan Yao, Yanyi Jiang, Longsheng Ma

**Affiliations:** State Key Laboratory of Precision Spectroscopy, East China Normal University, China; State Key Laboratory of Precision Spectroscopy, East China Normal University, China; State Key Laboratory of Precision Spectroscopy, East China Normal University, China

Frequency can be measured much more precisely than any other physical quantity, and much of precision metrology depends critically on the measurement of frequency and its inverse, time-interval. Optical electromagnetic waves (or ‘light’), oscillating at more than 10^14^ per second, are at least 1000 times more precise for time/length measurement compared to microwave frequencies near 1 GHz. However, electronics do not respond to such rapid optical oscillations. Thus, optical frequencies cannot be measured directly, and must be divided down to the radiofrequency (RF) regime for electronic counting. It has been a historical challenge to realize optical frequency division with arbitrary divisors. In the 1970s, the absolute frequency of a methane-stabilized He-Ne laser was measured by dividing its frequency to the RF region with a frequency chain based on many nonlinear frequency conversions. By measuring the laser frequency, the measurement accuracy of the velocity of light was improved 100 times [1]. However, the frequency chain for optical frequency division was complex and huge in size, and covered only a few specific wavelengths. The accuracy of the frequency chain was only 10^−10^, limiting its application in precision measurements with high accuracy.

Optical frequency combs, invented in 1999, have revolutionized the art of optical frequency division [2,3]. The spectrum of an optical frequency comb consists of a series of discrete optical frequency lines uniformly spaced by a RF interval. Using the comb to interfere with optical waves, researchers can link optical-optical and optical-microwave frequencies in a single step. In 2001, the first frequency comparison between a Ca optical clock and a Hg^+^ optical clock was made using an optical frequency comb [4]. In 2004, an international frequency comparison of four combs (two different types) from three laboratories verified that optical frequency combs could support optical frequency division with 10^−19^ uncertainty [5], confirming that combs can serve as the clockwork for optical atomic clocks with 10^−18^ uncertainty. Development of optical atomic clocks bloomed in the following 15 years: both the fractional frequency instability (long term) and uncertainty of optical clocks reached 10^−18^, and progress is being made towards 10^−19^–10^−20^ [6].

In most early work, the noise from combs, RF electronic timebases and other technical noise limited the division uncertainty at the 10^−19^ level. In 2015, most of the noise from combs and RF timebases was reduced using a transfer oscillator scheme [7,8] and synchronously counting relative to a H-maser. With these techniques of noise reduction, the frequency ratio of two independent frequency-stabilized lasers was simultaneously and independently measured with a Ti: sapphire comb and a fiber comb, and the agreement between these frequency ratio measurements was 3 × 10^−21^ [8]. In 2016, the comb frequency noise was further reduced by employing both the transfer oscillator scheme and a narrow-linewidth, frequency-stabilized comb. Meanwhile, a self-referenced RF timebase divided from one of the laser frequencies was introduced to remove the noise from an additional RF timebase. Along with noise reduction of common optical-path propagation, the uncertainty of optical frequency division was demonstrated to be 1.4 × 10^−21^ by comparing against the frequency ratio between the fundamental and the second harmonic light [9]. Additional laser linewidth broadening from optical frequency division was less than 1 mHz.

**Figure 1.**
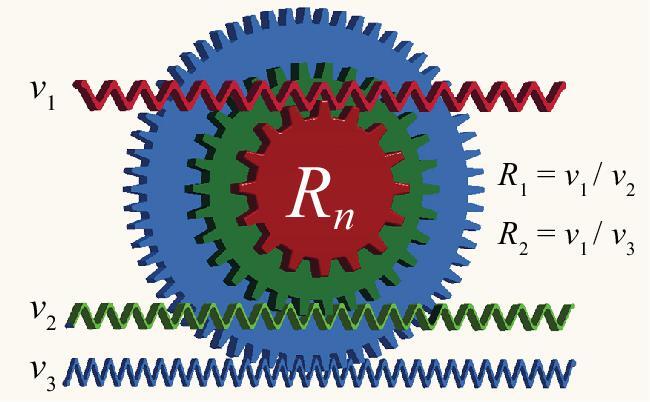
Multiple-channel optical frequency divider based on an optical frequency comb connects optical waves with different frequencies, behaving like gears. It can convert optical frequencies with pre-determined ratios, or measure optical frequency ratios.

The unprecedented precision and accuracy provided by optical clocks have led to eight optical frequency standards being recommended as ‘secondary representations of the second’, and a roadmap towards the redefinition of the SI second has been proposed [10]. To assure continuity between the present definition and the new definition, frequency comparisons (at least three) between optical clocks and Cs primary clocks (called absolute frequency measurements) should be made with uncertainties below 3 × 10^−16^. Moreover, to show the achievable accuracy provided by optical clocks beyond that from the present definition, frequency ratio measurements between optical clocks based on different species or transitions (at least five) with agreement below 5 × 10^−18^ should be performed. Even after the SI second redefinition, frequency comparisons between optical clocks will be required to measure the frequency instability, uncertainty and reproducibility of optical clocks. To meet the requirement of the SI second redefinition, accurate optical frequency dividers with multiple channels as shown in Fig. 1 must be developed for regular frequency measurement and transfer. If optical frequency dividers connect optical clocks with 1.5 μm lasers for signal transmission in optical fiber, together with fiber noise cancellation, an optical clock network will be established for time/frequency dissemination, geodesy and astronomy.

Other applications of optical frequency dividers with multiple channels include tests of fundamental physics measuring frequency ratios among different optical clocks at different times and locations. Do fundamental constants, such as the fine-structure constant and the proton-to-electron mass ratio, vary with time and space? Measurements of these dimensionless fundamental constants play a special role in tests of the equivalence principle. Moreover, using a multiple-channel optical frequency divider to accurately set the frequency ratios between an optical/RF frequency standard and wavelength-tunable, single-frequency continuous-wave lasers with useful power, coherent laser light can be generated at any desired wavelength over a wide optical region with high precision on demand, making the dreams of optical frequency synthesizers come true. Optical frequency synthesizers are invaluable for atomic and molecular spectroscopy, optical communications and coherent light detection. Moreover, they can also divide the frequency of optical clocks to the microwave region to take advantage of the accuracy and low phase noise provided by optical clocks.

Motivated by the above applications, researchers seek to reduce the noise and uncertainty of optical frequency dividers below that of optical clocks by at least one order of magnitude. Although accuracy is very important, to meet practical applications, optical frequency dividers also need to be improved in terms of compact size, continuous long-term operation and even portability. Ultimately, optical frequency dividers will be integrated on a photonic chip [11], similar to chip-scale digital RF frequency dividers. Also, the spectrum covered by optical frequency dividers needs to be extended to ultraviolet and to mid-infrared for wide applications. Looking to the future, optical frequency dividers have the potential for wide use in the SI second redefinition and precision measurements, thus they must be optimized for high accuracy and convenient use.
